# Nanostructures as Targeted Therapeutics for Combating Oral Bacterial Diseases

**DOI:** 10.3390/biomedicines9101435

**Published:** 2021-10-10

**Authors:** Shima Afrasiabi, Nasim Chiniforush, Hamid Reza Barikani, Alireza Partoazar, Ramin Goudarzi

**Affiliations:** 1 Laser Research Center of Dentistry, Dentistry Research Institute, Tehran University of Medical Sciences, Tehran 1441987566, Iran; shafrasiabi@alumnus.tums.ac.ir (S.A.); n-chiniforush@sina.tums.ac.ir (N.C.); 2 Dental Implant Research Center, Dental Research Institute, Tehran University of Medical Sciences, Tehran 1441987566, Iran; hrbarikani@tums.ac.ir; 3 Experimental Medicine Research Center, Tehran University of Medical Sciences, Tehran 1441987566, Iran; 4 Division of Research and Development, Pharmin USA, LLC, San Jose, CA 95128, USA

**Keywords:** nanoparticles, zinc oxide, biofilms, dental caries, root canal therapy, periodontitis, antimicrobial photodynamic therapy, drug delivery systems

## Abstract

Pathogenic oral biofilms are now recognized as a key virulence factor in many microorganisms that cause the heavy burden of oral infectious diseases. Recently, new investigations in the nanotechnology field have propelled the development of novel biomaterials and approaches to control bacterial biofilms, either independently or in combination with other substances such as drugs, bioactive molecules, and photosensitizers used in antimicrobial photodynamic therapy (aPDT) to target different cells. Moreover, nanoparticles (NPs) showed some interesting capacity to reverse microbial dysbiosis, which is a major problem in oral biofilm formation. This review provides a perspective on oral bacterial biofilms targeted with NP-mediated treatment approaches. The first section aims to investigate the effect of NPs targeting oral bacterial biofilms. The second part of this review focuses on the application of NPs in aPDT and drug delivery systems.

## 1. Introduction

Biofilms have an important impact on humans in many ways as they can develop in natural, medical, and industrial environments [1]. The oral cavity is an ideal environment for microbial cell growth, survival, and stability, followed by oral biofilm formation on the tooth surface. Biofilms contain a set of bacteria that are produced in extracellular polymeric substances (EPS). Bacterial growth results in the conversion of bacterial biofilm from commensal plaque to a pathogenic form. Bacteria present in the biofilm are significantly less sensitive to antimicrobial agents than planktonic bacteria [2]. In addition, biofilm-associated cells differ in physiology and trigger different gene expression profiles to adapt to the biofilm environment [3]. For example, the genes SMU.629 and SMU.1591 encode superoxide dismutase and catabolite control protein A, which are involved in the oxidative stress tolerance and the expression of biofilm-associated genes in *Streptococcus mutans*, respectively. SMU.629 and SMU.1591 were impressively increased in biofilm [4,5].

The bacterial composition of dental biofilms is relatively stable and mainly consists of sites that are protected from biofilm-removing forces applied in the mouth [6]. Biofilms formed on the teeth produce diseases such as caries, chronic gingivitis, and periodontitis that have a wide range of side effects. If dental biofilms persist, they become harder and form calculus in the lower and upper gum areas, which cause an inflammatory response of the host and increase the amounts of tissue fluid flowing into gingival pockets and can lead to gum disease [7].

The inhibition of oral bacterial biofilms is challenging. The low pH milieu indicative of oral biofilms reduces the efficacy of antibiotics on oral biofilms. The acidic environment induces further EPS synthesis. The presence of EPS reduces drug access and triggers bacterial tolerance to antibiotics [8]. The mechanical removal of bacterial biofilms from dental surfaces is a proven method of treating periodontal diseases. However, mechanical procedures alone are not able to entirely kill all bacteria. Furthermore, the use of metal instruments to remove oral biofilms will result in tooth structure removal over time, and can ultimately lead to gum resorption and tooth sensitivity to physical and thermal stimulation-induced effects [9]. It has also been reported that chlorhexidine has many adverse effects ranging from mild symptoms such as headache and dry mouth to severe symptoms such as tooth staining, calculus formation, and upper respiratory tract infection and, therefore, is not suitable for daily, long-term use. Alternative anti-biofilm agents include alcoholic mouthwashes, which can irritate the oral mucosa and lead to oral cancer [10]. Additionally, natural therapeutic drugs have little efficacy due to poor drug solubility, and low penetration of the EPS matrix [8].

An alternative way for infection control has been the design and development of novel biocompatible and non-absorbable nanoparticles (NPs), which may reach high local bioactivity in a controlled release of antibacterial effects [11]. NPs have distinct advantages in that they exhibit special physical and chemical properties due to their ultra-small sizes and large surface area-to-mass ratio. This includes increased reactivity, greater solubility, biomimetic features and the ability to be functionalized with other substances such as drugs, bioactive molecules and photosensitizers. Furthermore, antimicrobial NPs can effectively infiltrate the oral biofilms, leading to the effective delivery of therapeutics and may help manage the use of antibiotics [12]. The large surface area and high charge density of NPs enable them to interact with negatively charged bacterial cells, causing enhanced antimicrobial activity, cell membrane damage, generation of reactive oxygen species (ROS), interference with cellular processes, proteins destruction, and, finally, cell death induced by DNA damage [13]. Despite several benefits, NPs have some disadvantages, such as toxicity, refraining from the physiological barrier, evading rapidly from the phagocytic cells, and mounting an immune response that should be carefully considered if they are used in living organisms [14].

Antimicrobial photodynamic therapy (aPDT), which combines a photosensitizer and a light source, has been suggested as an alternative technique for bacteria inactivation. To perform efficient aPDT, photosensitizers must be able to infiltrate bacteria [15]. NPs improve the effectiveness of the photosensitizer because they can pass through the bacteria cell walls [16]. Additionally, nanotechnology in the case of drug delivery vehicles can improve drug availability into the target tissue to deliver the maximum therapeutic effect [17]. Herein, this review investigated the antimicrobial actions of NPs against oral infectious disease with a focus on oral bacterial biofilms. Additionally, we will also elaborate on the role of NPs when applied in aPDT and drug delivery systems.

## 2. Methods

An electronic search through Scopus, PubMed, Web of Science and Google Scholar databases were conducted. All databases were searched from their inception to 15 September 2021. The following terms were searched in combination: nanoparticles, biofilms, dental caries, root canal therapy, periodontitis, antimicrobial photodynamic therapy, and drug delivery systems. Then, full-text articles were read for a complete assessment and determination of inclusion or exclusion. The inclusion criteria for our review were all types of articles published in English involving the keywords.

## 3. NPs and Their Role in Dental Caries Control

Dental caries is a multifactorial and biofilm-mediated oral disease [18]. The basic mechanism of dental caries is an ecological shift within the dental biofilm to acidogenic bacteria growth, frequently made by exposure to fermentable carbohydrates. The change in pH leads to an imbalance between de/remineralization processes, thus creating clinical decay [19]. *S. mutans* is the major bacterial species of dental caries [20], and it is capable of reducing environmental pH that results in the decalcification of the tooth [20]. Controlling dental caries can, therefore, be achieved through preventing the bacterial action, helping reverse tooth demineralization, and promoting the remineralization process [21]. The complete removal of carious dentin before placing a restoration often results in a pulpal exposure [22]. According to scientific evidence, every effort ideally should be made to maintain viable pulp tissue [23]. The use of nanostructures in dental caries management has also received much attention [24]. One study explored how chitosan (CS) combined with zinc oxide/zeolite (ZnO/Z) nanocomposite accelerates the biofilm reduction, metabolic activity and cariogenic properties of *S. mutans* [25]. The effect of ZnONPs on bacteria is through damage to the cell wall, lipids and protein, resulting in the leakage of intracellular contents to the outside of the bacteria and, ultimately, bacterial death [26].

Furthermore, recurrent caries is a major factor for restoration failure [27]. To address this problem, an adhesive containing NPs of amorphous calcium phosphate (NACP) was developed. NACP could enhance the Ca and P ions released under low pH conditions. Moreover, NACP adhesive could rapidly change the solution pH from 4.0 to above 5.5, improving the dentin remineralization. The NACP adhesive was also reported to show inhibition properties against bacteria and decrease the colony-forming units count in a biofilm of *S. mutans* [28].

Furthermore, metallic NPs are popular due to their antibacterial properties and biocidal activities at low concentrations. Copper NPs (CuNPs) and ZnONPs have been demonstrated to be effective against a wide range of bacteria. The addition of CuNP/ZnONP in an adhesive system provides more antimicrobial activities, without affecting their bond strength. Moreover, CuNP/ZnONP also inhibited matrix metalloprotease-2, and may improve enamel remineralization [29]. Matrix metalloprotease-2 is associated with the development of dental germ as well as the progression of dental caries in patients with necrotic pulp [30].

Another pertinent example is that of Cao and colleagues, who used a novel resin-based dental composite containing a photocurable core–shell of silver bromide (AgBr) plus cationic polymer nanocomposite (AgBr/BHPVP). The potent antibacterial effects of this resin composite against *S. mutans* are exerted through its biocide-releasing mechanism of the Ag^+^ ions. Thus, resins containing AgBr/BHPVP NPs with long-term antimicrobial effects exhibited excellent potential as an antimicrobial agent which could be used in dental products [31].

## 4. NPs and Their Role in Root Canal Treatment

The failure of root canal treatment is attributed to the eradication of bacteria and incomplete disinfection of the complex root canal system, which will inevitably lead to persistent apical periodontitis. *Enterococcus faecalis* is a bacterium often isolated from the root-filled teeth with chronic apical periodontitis [32]. *E. faecalis* invades the dentinal tubules, adheres to the root canal wall and forms a biofilm on dentin [32]. Endodontic diseases are primarily associated with biofilm-mediated infection [33]. Microbial biofilms inside the root canal are very resistant to conventional medicaments including systemic antibiotics and sealers. The anatomical complexities and the multi-species biofilms increase the difficulty in the complete eradication of *E. faecalis* microbial biofilm. In recent years, the application of NPs to disinfect root canals has gained attention due to their broad spectrum antibacterial activity [34].

A study investigated the antibacterial activity of propolis NPs (300 μg/mL) with an average size of 117.6 nm as a root canal irrigant against a root canal infected with *E. faecalis* biofilm. Results confirmed propolis NPs were equally effective as NaOCl (6%) and chlorhexidine (2%) in reducing the *E. faecalis* biofilm [35]. Interestingly, an in vitro study exhibited promising results on root canal surfaces treated with cationic antibacterial NPs such as ZnONP, CS/ZnONP, or CS-layer-ZnONP. But this effect was not considered efficient enough to elicit whether the inhibition of bacterial recolonization and biofilm formation was due to the killing of bacteria or by the direct effect of NPs on the bacteria–substrate interaction [36].

Recently, a novel antibacterial root canal sealer (dimethylaminohexadecyl methacrylate (DMAHDM)), and NACP were developed and tested on *E. faecalis* biofilm inhibition. There was a 3-log reduction in *E. faecalis* counts due to the new sealer compared to the control. Moreover, this root canal sealer demonstrated acid-neutralizing capabilities which could be useful in preventing anaerobic growth. The therapeutic root canal sealer, by releasing Ca and P ions, was able to inhibit *E. faecalis* biofilms. This structure, with antibacterial effects and remineralization capabilities, is a promising strategy to improve the success rate of endodontic therapy and dentin hardness [37].

## 5. NPs and Their Role in Periodontitis and Peri-Implantitis

Periodontitis, a chronic inflammatory condition, usually gradually destroys the tooth-supporting structures, subsequently leading to the looseness and, finally, the loss of teeth. Periodontitis is not only considered a major risk of further tooth loss, but is also reported to be associated with systemic disorders [38]. Periodontitis is one of the main risk factors that enhance the risk for peri-implantitis [39]. Peri-implantitis is an inflammatory disease affecting the tissues surrounding osseointegrated dental implants resulting in pocket formation, purulence and the loss of supporting bone, which is associated with the reduction of implant survival [40,41]. The accumulation of bacteria on the surface of implant and multi-species biofilm formation is considered the cause of dental implant failure and peri-implantitis [41].

*Porphyromonas gingivalis*, *Prevotella intermedia* and *Aggregatibacter actinomycetemcomitans* are the top three periodontitis and peri-implantitis-associated species in subgingival plaque. In the periodontal pockets, these bacteria can regulate the expression of many virulence factors that lead to supporting bone loss [42]. Recent studies have recently stated significant transcriptional changes when the above-mentioned bacteria were growing within a microbial multi-species biofilm [43,44,45]. Despite comprehensive mechanical and antimicrobial treatment, the long-term control of infection is impossible [46]. The use of NPs in the periodontal field is fascinating, particularly for infection control and oral biofilm management [47].

Carbon quantum dots are a new type of carbon-based nanomaterials which have unique properties such as high photostability, favorable water solubility, biocompatibility, low toxicity, and ease of preparation and modification. Liang and colleagues fabricated tinidazole carbon quantum dots using the hydrothermal method. The antimicrobial activity of tinidazole carbon quantum dots depends on its ability to impair toxicity by inhibiting the main virulence factors associated with the biofilm formation of *P. gingivalis* and, hence, affecting the self-assembly process of biofilm-related proteins [48].

Nanoscale surface modification has been suggested to affect bacterial adherence and biofilm formation on implants. Besinis et al. confirmed that a dual-layered silver plus nano-hydroxyapatite (HA) coating exhibited a strong inhibitory effect on the growth of *Streptococcus sanguinis* in the surrounding media and reduced the biofilm on the titanium-based implant surface by 97.5% [49]. *S. sanguinis* plays a significant role as the initial colonizer in the early adhesion stage of biofilm formation and titanium implant colonization, because it bonds directly to the implant surface and facilitates the later bacterial adhesion [50]. This novel nanocoating on titanium implants decreased infection risk, improved osseointegration, and enhanced bone healing [49].

## 6. NPs and Their Role in Orthodontics Infection Control

Fixed orthodontic treatments hamper the proper cleaning process, leading to biofilm buildup and white spot lesion formation. After orthodontic therapies with fixed appliances, there are significant increases in the amounts of cariogenic bacteria and biofilm formation [51]. Adding NPs to the orthodontic adhesives can reduce the frictional force between the orthodontic bracket and wire, improve the antimicrobial properties, and prevent enamel demineralization during orthodontic treatment [52].

The inhibitory effect of silver NPs in biofilm growth could predict that the silver NPs reduce biofilm metabolic activity [53]. Additionally, Xie et al. reported that the dental composite containing NACP considerably inhibited enamel demineralization and white spot lesions around orthodontic brackets [54]. Another study showed that orthodontic composites containing 1% titanium oxide NPs could significantly reduce the number of bacterial cells compared with conventional composites, without compromising the shear bond strength [55].

## 7. NPs and Their Role in Denture Base Material

The use of removable partial and complete dentures acts as a factor that favors bacterial accumulation in the oral cavity. The use of denture base material that possesses an antimicrobial effect may prevent future recurring infections by managing microbial colonization and halting biofilm formation [56]. Poly (methyl methacrylate) (PMMA) has been used as a principle polymer-based material for dentures. Titanium dioxide NP with antimicrobial properties against a wide range of bacteria has been recommended as filler in polymeric materials [57]. Anehosur and co-workers found that PMMA containing 3% titanium dioxide NP decreased the amount of *Staphylococcus aureus*, prevented quorum sensing, and eventually stopped biofilm formation on denture surface [58]. Likewise, Bacali and co-workers investigated the addition of graphene silver NP to PMMA acrylic resins in an attempt to improve denture antimicrobial characteristics and found that this combination restricted the growth of *P. gingivalis* and *E. faecalis*. In addition, the use of extra aPDT showed a greater effect on the growth inhibition of these two bacterial strains [59]. A summary of NP applications developed against oral bacterial infections are listed in Table 1.

## 8. Applications of NPs in aPDT

**Figure 1:**
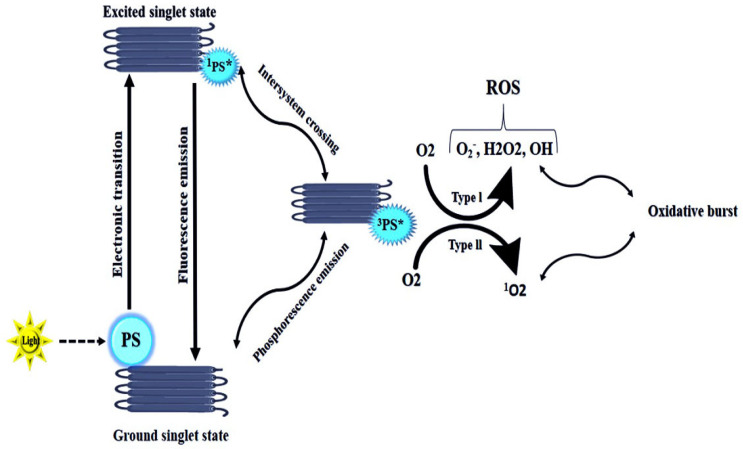
Mechanism of action of antimicrobial photodynamic therapy. The ground state (S0) photosensitizer absorbs a photon to form the first excited singlet state (^1^PS) that can undergo intersystem crossing to form the first excited triplet state (^3^PS). The long−lived triplet state can interact with molecular oxygen in two ways: type I electron transfer to form superoxide and the hydroxyl radical, or type II energy transfer to form ^1^O2. The generated ROS can damage a broad spectrum of microorganisms.

Antimicrobial photodynamic therapy (aPDT) has become an attractive alternative to traditional treatment thanks to outstanding advantages such as its harmless visible light, ease of use, and, above all, its sensitivity to ROS-mediated killing in microorganisms [81]. The employment of aPDT involved a light source under a specific wavelength in combination with photosensitizers and oxygen, which allowed an attack against microorganisms by producing ROS. The singlet photosensitizer in the ground state was extremely unstable and could instantaneously release energy, generating the triplet state. The released energy was absorbed by the tissue oxygen to form ROS, which had strong oxidative stress and cell death [82] (Figure 1).

Studies have found that aPDT exhibits an efficient bactericidal performance against oral bacteria [83,84,85,86,87]. Fumes et al. investigated aPDT’s effect on *S. mutans* using methylene blue (MB) as the photosensitizer and an energy density of 6 J/cm^2^. The results showed that aPDT can effectively decrease the viability of *S. mutans* biofilms [84]. Goulart et al. examined the efficacy of aPDT by using MB and erythrosine and an energy density of 0.65–2 J/cm^2^ to inactivate *A. actinomycetemcomitans*. The decrease in the *A. actinomycetemcomitans* biofilm in the presence of MB and erythrosine was 54% and 77%, respectively [85]. Street et al. demonstrated that the ability of aPDT, by using MB and an energy dose of 9.4 J/cm^2^, in eradicating the biofilm form of *Fusobacterium nucleatum*, *P. gingivalis*, and *A. actinomycetemcomitans* [86]. López-Jiménez et al. also demonstrated the effect of aPDT using MB or toluidine blue O (TBO) with an energy density of 271 and 106.4 J/cm^2^, respectively, on disrupting *E. faecalis* biofilms [87].

**Figure 2:**
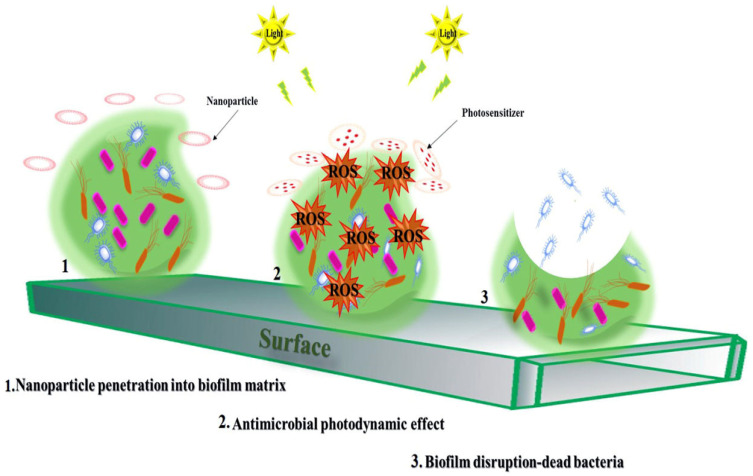
Schematic presentation of antimicrobial photodynamic therapy with nanoparticles against oral biofilm. Nanoparticles can accumulate on the membrane surface leading to local deformation and, ultimately, damaging membrane integrity. Photosensitizer-loaded nanoparticle-guided synergistic antimicrobial photodynamic therapy. After entering into the bacteria in the target site, ROS can be effectively produced under the irradiation as shown in the figure. This mechanism can improve the antimicrobial photodynamic therapy function.

Nevertheless, the drawbacks of conventional photosensitizers, including poor solubility in aqueous media, limited in vivo stability, low extinction coefficient, and poor target selectivity, had hampered the clinical applications. To overcome these challenges, nanomaterials have been shown to be highly efficient and safe when complexed with photosensitizers. They possess important properties: controlled release of photosensitizers, enhanced water solubility, the ability to prevent aggregation even at high concentrations, excellent binding, improved ROS production, and increased bacterial cell wall penetrability [88] (Figure 2). Furthermore, nanosystems can deliver photosensitizers into cells and protect them from light, temperature, pH, and enzymatic degradation, and can decrease high-energy doses of light as well as the frequency of the administration of photosensitizers [89]. In recent years, NP-based aPDT has become a hot topic in antibacterial therapy [42].

In previous work by our group, propolis NPs in combination with PhotoActive^+^ and TBO as photosensitizers indicated higher antibacterial activity during aPDT against *S. mutans* than the individual photosensitizer. Furthermore, the expression level of genes that play an important role in *S. mutans* biofilm formation was also decreased to approve the high efficiency of the proposed NPs in combination with photosensitizers [90].

In another study, aPDT with MB-poly(lactic-co-glycolic) [PLGA] cationic NPs (50 µg/mL and 30 J/cm^2^) in chronic periodontitis patients reduced bacterial viability in both planktonic and biofilm lifestyles by approximately 60% and 48%, respectively [91]. Perhaps it can be concluded that the MB-NP was able to diffuse MB dye within biofilms [92]. A summary of NP applications in aPDT against oral bacterial infections are listed in Table 2.

## 9. Application of NPs in Drug Delivery Systems

The effective drug concentration reached within the biofilm is very crucial [17]. The impaired diffusion of antimicrobial agents in the biofilm mode of growth is another cause of antimicrobial therapy failure. Restricted penetration of the drug is, on the one hand, caused by cell–cell interactions within the biofilm matrix and, on the other hand, bacterial cells that exist in a dormant state and are organized into structured communities embedded within an extracellular matrix [100].

Currently, nano-scale drug delivery systems have emerged as attractive platforms. They could improve therapeutic effects and decrease the unwanted after-effects compared with conventional medicine [101]. Nanocarriers should be able to encapsulate drugs efficiently, while having the ability to increase drug transport to the deep biofilm structure [100]. One example is ciprofloxacin (CIP) in biofilms related to root canal infections. In a recent study, antibacterial and anti-biofilm activity of CIP-loaded PLGA NPs and CIP-PLGA NPs coated with CS (CIP-PLGA/CS) versus CIP solution as a control on *E. faecalis* was evaluated. CIP-PLGA/CS showed lower release (78.3%) than the CIP in the first hour (100%). CIP-PLGA/CS showed the highest decrease in biofilm inhibition (86.4%) which indicated that CS NPs are able to penetrate the biofilm and promote antibacterial effects. In addition, CIP-PLGA/CS, dramatically declined the count of *E. faecalis* strains in all concentrations [32].

The drug delivery systems in periodontal pockets had a beneficial therapeutic application for periodontitis [101]. In one study, antibacterial activity of the free minocycline and minocycline-PEGylated PLGA NPs were examined against *A. actinomycetemcomitans*. The results demonstrated that the encapsulation of minocycline into the PEGylated PLGA NPs increased the antibacterial efficiency against *A. actinomycetemcomitans* 2-fold, compared to that of the drug alone. This was possibly due to an increase in the penetration of the NPs into the microbial community and a better delivery of minocycline to the target cells [102]. Similarly, tetracycline-loaded titanium dioxide nanotube revealed excellent antibacterial and anti-adherence effects against *P. gingivalis* [103].

Nanocarriers can increase the beneficial effects of aPDT by increasing stability, enhancing the selectivity of treatment and inhibiting aggregation known to reduce the phototoxicity commonly seen in the treatment. The nonspecific distribution of the photosensitizer after administration represents a major obstacle to drug efficacy in aPDT. In addition, some of the photosensitizers are degraded in photolytic and hydrolytic stress conditions or metabolized after administration [104].

The results of a study showed nanocarriers with enhanced antibacterial effects could improve the efficiency of photosensitizers. The efficacy of a photosensitizer loaded with various nanocarriers including GO, GO-carnosine (Car) and GO-Car/HA were investigated against *S. mutans* via irradiation with an 810 nm diode laser system [105]. Graphene oxide (GO), a one atom thick molecule, easily adsorbs polymers and proteins, negatively interfering with microorganisms’ colonization, aggregation and their initial adhesion to surfaces. These interesting, recognized GO properties represent a promising approach for antimicrobial and anti-biofilm applications [106]. GO-indocyanine green (ICG), GO-Car-ICG and GO-Car/HA-ICG-mediated aPDT significantly declined the count of *S. mutans*. They also reduced biofilm formation by more than 50%. The expression of the gtfB gene was considerably reduced several-fold after aPDT was in the presence of the formulation. It could be concluded that the GO exhibited great potential to yield high stability and increase the inhibitory effects of ICG-mediated aPDT against *S. mutans* [105].

Hydrogel-based drug delivery systems have also received considerable attention [107]. NP-loaded hydrogels can not only enhance the bioavailability, efficacy and safety of the drugs, but also achieve higher mechanical strength and extend the drug in the target tissue [108,109]. Recently, Johnson et al. developed an antimicrobial-loaded nanofiber hydrogel-based drug delivery system composed of cellulose nanofibers and κ-carrageenan oligosaccharides NPs for the treatment of periodontitis. This material has strong antibacterial activity against periodontal pathogens. It has been reported that this material helps to reduce ROS generation and the cytokines production level under inflammatory situations [107].

## 10. Conclusions and Perspectives

The human oral cavity has been found to be an environment with bacterial diversity. In line with this, dysbiosis in the oral cavity is linked with many diseases such as dental caries, root canal infection, and periodontal disease. Overall, it can be said that the NPs are a valid therapeutic modality that can feasibly be implemented in the oral healthcare system. The potentiation of aPDT by NPs is one such candidate for effective antimicrobial therapies, as demonstrated in this review. The scope of future research lies in the development of NP technologies for the therapeutic management of oral infections and the evaluation of models to re-evaluate the removal of the biomass from the teeth, in addition to assessing the action of novel antimicrobial agents on multiple-species biofilm. The scarcity of clinical studies highlights the need for more in vivo investigations. Thus, we recommend more regular steps to be taken in order to put the nanostructure-based formulations that have been proposed on the market.

## Figures and Tables

**Table 1:** A summary of several NPs developed for combating oral bacterial infections.

Nanoparticles	Size (nm)	Microorganism	Nanoparticle Therapeutic Effect	Ref.
Cerium oxide	-	*S. mutans*	- Reduces adherent bacteria	[60]
Magnesium oxide	~20	*S. mutans* *S. sobrinus*	- Antibacterial and anti-biofilm activity	[61]
Polyethyleneimine	-	*S. mutans*	- Prevents caries and inflammation	[62]
NACP	~116	Total *streptococci*	- Increases the Ca and P ion release at cariogenic pH - Inhibits biofilms and remineralizes tooth lesions - Reduces metabolic activity and lactic acid of biofilm	[63]
ZnO and silver	~50 and 20	*S. mutans* *Lactobacillus*	- Inhibits bacterial growth	[64]
Graphene	-	*S. mutans*	- Inhibits *S. mutans* adhesion and growth - Anti-biofilm effects - Application as filler in dental adhesives	[65]
MSN-PLGA	~107	*S. mutans*	- Anti-biofilm effects - Application as adhesive and restorative dentistry	[66]
ZnO/Z	~30	*E. faecalis*	- Anti-biofilm effects - Decreases esp gene expression	[67]
AgNPs-PL	~21.62	*E. faecalis*	- Increases their permeability - Destabilizes bacterial membranes - Anti-biofilm activity	[68]
Bismuth NPs	~40	*E. faecalis*	- Antibacterial activity	[69]
CO and CH	-	*E. faecalis*	- Antibacterial activity	[70]
MgO	~70–150	*E. faecalis*	- Antibacterial activity	[71]
Chitosan	~85–221	*E. faecalis*	- Anti-biofilm activity - Inhibition of bacterial recolonization	[72]
Ag/Zn-MCSNs	~200–250	*E. faecalis*	- Anti-biofilm activity - Destroys cell membranes	[73]
Iron oxide	-	*E. faecalis*	- Improves catalytic activity - Enhances antibacterial activity on root canal surfaces	[74]
Chitosan-propolis	~247–512	*E. faecalis*	- Disrupts the biofilm structure - Alters the expression of biofilm-associated genes	[75]
Selenium	~40–150	*E. faecalis*	- Anti-biofilm activity	[76]
PLLA@Ag	-	*S. aureus*	- Antibacterial activity - Good biocompatibility - Implant-associated infections prevention	[77]
AgCSP	~300	*P. gingivalis*	- Antibacterial activity - Good biocompatibility - Tissue regeneration	[78]
AgNP/NSC	-	*A. actinomycetemcomitans*	- Kills the adherent bacteria - Anti-biofilm activity - Control of peri-implantitis	[79]
Bismuth subsalicylate	~4–22	*P. gingivalis* *A. actinomycetemcomitans*	- Antibacterial activity - Application in dental materials	[80]

NACP: amorphous calcium phosphate NPs, MSN-PLGA: mesoporous silica nanoparticles (MSN) grafted with poly-L-glycolic acid (PGA), ZnO/Z: zinc oxide/zeolite nanocomposite, AgNPs-PL: silver nanoparticles poloxamer thermoreversible gel, CO: calcium oxide, CH: calcium hydroxide, MgO: nano-magnesium oxide, Ag/Zn-MCSNs: silver and zinc incorporated mesoporous calcium-silicate nanoparticles, PLLA@Ag: silver nanoparticle-loaded poly(L-lactide), AgCSP: chitosan/polyurethane (CSP) nanofibrous membrane incorporated silver nanoparticles, AgNP/NSC: silica-based composite coating containing starch-capped silver nanoparticles.

**Table 2:** A summary of nanoparticle applications in aPDT against oral bacterial infections.

Nanoparticles	Size (nm)	Photosensitizer	Light Source	Microorganism	Therapeutic Effects	Ref.
Nano-c	-	ICG	810 nm light at an energy density of 4–24 J/cm^2^	*P. gingivalis*	- Bactericidal effects	[93]
Chitosan	~60	Rose bengal	260 nm light at an energy density of 4–60 J/cm^2^	*S. oralis*, *P. intermedia*, *A. naeslundii*	- Increased affinity to bacterial cell membrane - Anti-biofilm effects against multispecies bacterial biofilms	[94]
Fe_3_O_4_	~122.4	Chlorin e6	630 nm light at an energy density of 100 J/cm^2^	*S. sanguinis* *F. nucleatum* *P. gingivalis*	- Anti-biofilm activity - Inhibits the occurrence and progression of periodontitis	[95]
Lys-Au	-	Rose bengal	LED	*S. mutans*	- Bacterial inactivation - Anti-biofilm activity	[96]
Silver	~18	TBO	630 nm light at an energy density of 100 J/cm^2^	*S. mutans*	- Anti-biofilm activity - Anti metabolic activity	[97]
HPMC	-	TBO	630 nm light at an energy density of 32.4 J/cm^2^	*S. aureus* *A. actinomycetemcomitans* *P. gingivalis*	- Anti-biofilm activity	[98]
UCNPs	~25	Chlorin e6	980 nm light at an energy density of 750 J/cm^2^	*P. gingivalis* *P. intermedia* *F.nucleatum*	- Anti-biofilm effects	[83]
Liposomes	~100–150	tetra-m-hydroxyphenyl chlorin	652 nm light at an energy density of 100 J/cm^2^	*E. faecalis*	- Antibacterial activity	[99]

Nano-c: nanospheres coated with chitosan, ICG; indocyanine green, *S. oralis*: *Streptococcus oralis*, Lys-Au NCs: lysozyme-stabilized gold nanoclusters, LED: light-emitting diode, HPMC: chitosan hydrogels containing hydroxypropyl methylcellulose, UCNPs: upconversion nanoparticles (UCNPs) NaYF4:Yb,Er.
